# Establishment of prognostic models of adrenocortical carcinoma using machine learning and big data

**DOI:** 10.3389/fsurg.2022.966307

**Published:** 2023-01-06

**Authors:** Jun Tang, Yu Fang, Zhe Xu

**Affiliations:** ^1^ Department of Pediatric Surgery, The First Affiliated Hospital of Sun Yat-sen University, Guangzhou, China; ^2^ Department of Pediatrics, China Medical University, Shenyang, China

**Keywords:** adrenocortical carcinoma, machine learning, SEER, BP-ANN, survival status

## Abstract

**Background:**

Adrenocortical carcinoma (ACC) is a rare malignant tumor with a short life expectancy. It is important to identify patients at high risk so that doctors can adopt more aggressive regimens to treat their condition. Machine learning has the advantage of processing complicated data. To date, there is no research that tries to use machine learning algorithms and big data to construct prognostic models for ACC patients.

**Methods:**

Clinical data of patients with ACC were obtained from the Surveillance, Epidemiology, and End Results (SEER) database. These records were screened according to preset inclusion and exclusion criteria. The remaining data were applied to univariate survival analysis to select meaningful outcome-related candidates. Backpropagation artificial neural network (BP-ANN), random forest (RF), support vector machine (SVM), and naive Bayes classifier (NBC) were chosen as alternative algorithms. The acquired cases were grouped into a training set and a test set at a ratio of 8:2, and a 10-fold cross-validation method repeated 10 times was performed. Area under the receiver operating characteristic (AUROC) curves were used as indices of efficiency.

**Results:**

The calculated 1-, 3-, 5-, and 10-year overall survival rates were 62.3%, 42.0%, 34.9%, and 26.1%, respectively. A total of 825 patients were included in the study. In the training set, the AUCs of BP-ANN, RF, SVM, and NBC for predicting 1-year survival status were 0.921, 0.885, 0.865, and 0.854; those for predicting 3-year survival status were 0.859, 0.865, 0.837, and 0.831; and those for 5-year survival status were 0.888, 0.872, 0.852, and 0.841, respectively. In the test set, AUCs of these four models for 1-year survival status were 0.899, 0.875, 0.886, and 0.862; those for 3-year survival status were 0.871, 0.858, 0.853, and 0.869; and those for 5-year survival status were 0.841, 0.783, 0.836, and 0.867, respectively. The consequences of the 10-fold cross-validation method repeated 10 times indicated that the mean values of 1-, 3-, and 5-year AUROCs of BP-ANN were 0.890, 0.847, and 0.854, respectively, which were better than those of other classifiers (*P* < 0.008).

**Conclusion:**

The model combined with BP-ANN and big data can precisely predict the survival status of ACC patients and has the potential for clinical application.

## Introduction

Adrenocortical carcinoma (ACC) is an uncommon malignancy whose annual incidence is around 0.5–2/1,000,000. The peak age of onset is children under 5 years and patients in the age group between 40 and 50 years (1). Limited treatment and the high rate of metastasis make the prognosis of ACC patients poor. The 5-year overall survival rate of ACC patients is between 15% and 60%, which is 54%–84% in stage I cases and 0%–18% in stage IV cases (2). Due to the diverse clinicopathologic characteristics of this condition, the prognosis of the patients can be different. Therefore, it is vital to identify patients at high risk for developing ACC to improve their survival rate. Meanwhile, the low incidence of ACC makes it difficult for a single medical center to collect sufficient cases for study. One of the solutions to this problem is the collection of data through the use of public databases. One of these databases is the Surveillance, Epidemiology, and End Results (SEER) database, which provides information on cancer statistics to reduce the cancer burden among the US population.

In recent years, several papers were published on ACC, in which the data were obtained from SEER. These articles applied similar clinical factors to establish Cox's proportional hazards regression models and fabricate nomograms (3–5). Among them, Kong et al. used more than 700 case records to build a Cox model and cases from the Cancer Genome Atlas (TCGA) database and multiple medical centers as external validation (4). Until now, the validation of this research is considered the most adequate. However, it may be for the sake of uniformity of prognostic factors among various datasets. The authors only included age and tumor-node-metastasis (TNM) stages in the prediction model, which limited the efficacy of the model. There are also other reports that added more predictive factors in their studies; however, these lack external data, decreasing the possibility of extrapolation.

Machine learning is a critical field of artificial intelligence. When conducting clinical studies, machine learning algorithms and clinical, imaging, and genomic information are often utilized to solve regression and classification problems. Yet, there is no research on using these algorithms combined with big data to establish machine learning-based (ML-based) models of patients with ACC. In this article, we attempt to employ four frequently used machine learning methods to construct forecasting models and compare their predictive efficiencies.

## Materials and methods

### Data collection and process

This is a retrospective study. Data of patients with ACC from the SEER database were used for the establishment and internal validation of models. Patients diagnosed with ACC between 1975 and 2018 were screened according to a series of criteria (mentioned below). Patients with primary ACC were retrieved from the location codes C74.0 – cortex of adrenal gland and C74.9 – adrenal gland and the International Classification of Diseases for Oncology-3 (ICD-O-3) morphology code 8370 – adrenal cortical carcinoma. The inclusion criteria are as follows: (1) patients with the location code of C74 or C74.9 and the ICD-O-3 morphology code 8370; and (2) patients diagnosed with ACC histologically. The exclusion criteria are as follows: (1) patients diagnosed with ACC based on only one of the following: symptoms, imaging, exfoliative cytological, or gross pathological evidence; (2) patients with incomplete follow-up data, including duration of follow-up and survival status; (3) unknown T or N stage; and (4) patients who died of causes other than ACC or suffered from other tumors simultaneously.

### Definition of forecasting variables and construction of models

Variables that might influence the survival of ACC patients were selected as candidates for factors to be included in the model, including gender, race, age, T stage, N stage, surgery, tumor size, and liver, lung, and bone metastasis. X-tile software was developed by Yale University to calculate the best cutoff values of continuous variables (6). The software was employed to group factors of age and tumor size. Therefore, the predictive factors included in our research were all discrete types. When structuring models for different time points, those who survived longer than corresponding time periods were excluded.

Construction of ML-based models requires both “training” and “testing” procedures. With respect to internal validation within the SEER dataset, the filtered patients were partitioned into training and test sets at a ratio of 8:2. Machine learning models were trained using the 80% part of ACC patients on the SEER registry and then the other 20% of data was used to test the predictive power of trained models. Furthermore, in case there was no algorithm performing the best statistically, the 10-fold cross-validation method repeated 10 times was performed. These functions to characterize the differences between models are based on the work of Hothorn et al. (7) and Eugster et al. (8).

Four common machine learning algorithms were tested to predict the survival status of patients, involving backpropagation artificial neural network (BP-ANN), random forest (RF), support vector machine (SVM), and naive Bayes classifier (NBC). The objective of this study was to explore the efficiency of forecasting the 1-, 3-, and 5-year survival status of patients with ACC via ML-based models, namely, “alive” and “dead.” Five-fold cross-validation was employed for parameter adjustment of all four ML algorithms. The classic metrics, area under the receiver operating characteristic (AUROC) curve, can represent model performance at every time point.

Approaches of parameter adjustment and construction of modeling methods and codes hailed from the official website page of R package “caret” (http://topepo.github.io/caret/model-training-and-tuning.html#customizing-the-tuning-process).

### Statistical analysis

Clinical data were downloaded from the SEER database by using SEER*Stat (8.3.9.2) software. All statistical analyses and data processes were performed by R (4.0.3) software. Figure processing was completed using Adobe Illustrator CC 2019. Models were trained and tested using the “caret” package. Two-sided *P* < 0.05 was thought to be statistically significant. The Kaplan–Meier method and log-rank test were performed to compare the survival status of patients with ACC in diverse groups. Comparison of means between two groups of continuous variables was carried out through a *t*-test, while Bonferroni correction was adopted for pairwise comparison of multiple principal averages. The AUROC curve represents the predictive efficiency of ML-based models.

## Results

**Figure 1:**
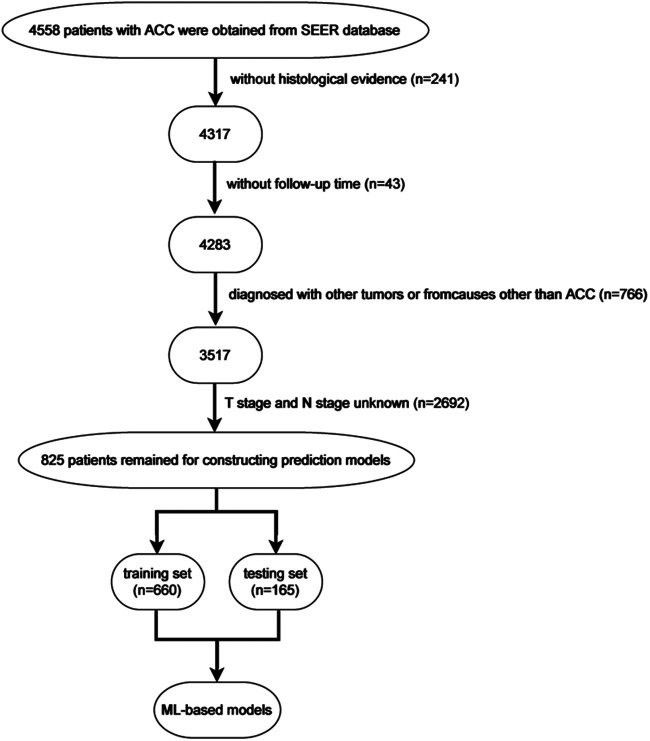
Flow chart showing the exclusion process of patients with ACC from the Surveillance, Epidemiology, and End Results (SEER) program database and internal and external validation.

**Figure 2:**
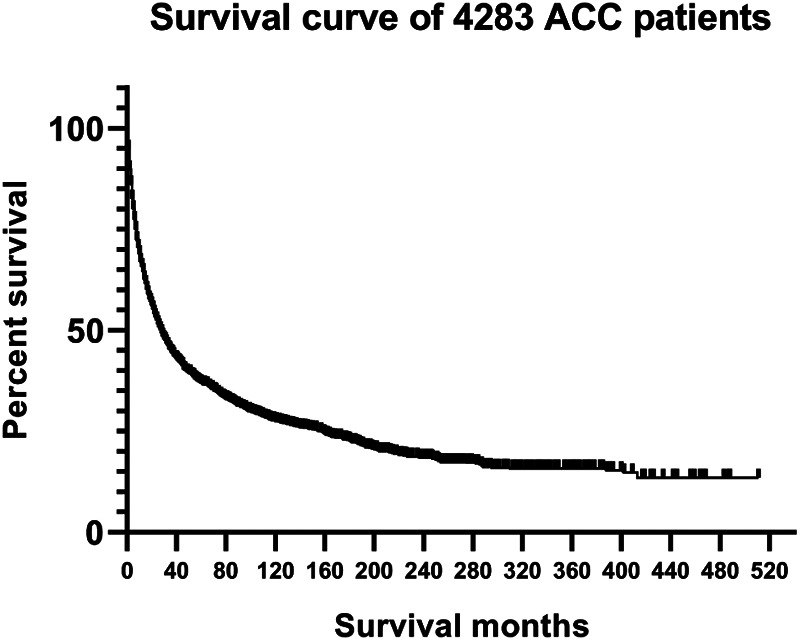
Overall survival curve of 4,283 patients with ACC.

From the SEER database, 6,206 patients in the subcategory of C74 or C74.9 were found and 8,370 patients from the ICD-O-3 morphology code were found; after screening, 825 patients were selected (Figure 1). Figure 2 shows the overall survival curve of the 4,283 cases diagnosed with ACC through histology with full follow-up data from the SEER program database. Table 1 and Figure 2 demonstrate the survival rate of these patients in each year; a declining trend of survival probability can be observed. Notably, the overall survival curve for the 4,283 ACC patients illustrated a steeply declined within the first 60 months; thereafter, the downward trend slowed down significantly. The 1-, 3-, and 5-year survival rates of ACC patients were 62.3%, 42.0%, and 34.9%, respectively. During the whole follow-up duration, 3,050 patients died as a result of ACC, accounting for 71.2% of total patients. The number of deaths in the first year was 1,583, accounting for 51.9% of the total deaths. The number of deaths during the first year was triple the number in the second year, which was 499 deaths. The number of deaths during the third year was 299. The total number of deaths in the first 5 years accounted for 85.7% of all deaths.

**Table 1:** Survival of 4,283 patients with ACC from the SEER database.

Follow-up (years)	Survival rate (%)
1	62.2
2	49.5
3	42.0
4	37.8
5	34.9
6	32.9
7	30.7
8	28.8
9	27.3
10	26.1

SEER, Surveillance, Epidemiology, and End Results.

**Figure 3:**
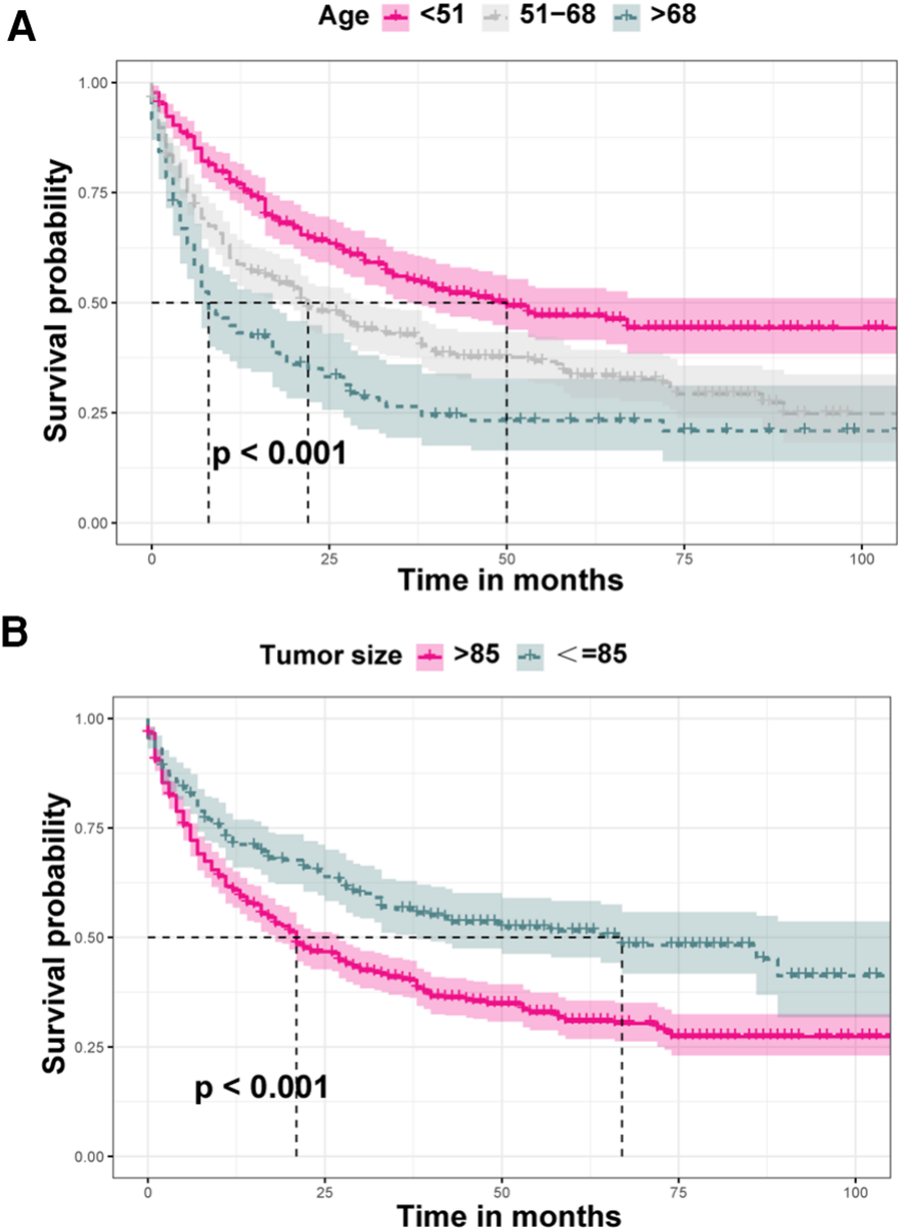
Comparison of diverse survival curves among various groups of age and tumor size. (**A**) Patients aged less than 51 years showed the highest survival rate, while patients aged above 68 years showed the worst survival rate, and the survival curve of patients aged 51–68 years was in the middle position; (**B**) survival probability of patients with tumors larger than 85 mm was lower that of those with tumors less than or equal to 85 mm.

Through loading data from the SEER dataset onto X-tile software, Kaplan–Meier survival analysis indicated that when age at diagnosis and tumor size were categorized into <51, 51–68, >68 years old and ≤85 and >85 mm, respectively, survival curves of each subgroup clearly split (Figure 3, *P* < 0.001). As shown in Table 2, Kaplan–Meier survival analysis showed significant differences among subgroups of age, T stage, N stage, surgery, tumor size, and liver, lung, and bone metastasis, according to the information retrieved from the SEER dataset. These demographic and clinical features were included in ML-based models as predictive variables.

**Table 2:** Description of the study population deriving from the SEER program database.

Features	SEER dataset (*n* = 825)	*P*-value
Sex		0.413
Female	513 (62.2)	
Male	312 (37.8)	
Age (years)		<0.001
<51	352 (42.7)	
51–68	352 (42.7)	
>68	121 (14.6)	
Race		0.192
Black	69 (8.4)	
White	683 (82.8)	
Other	73 (8.8)	
Laterality		0.391
Left	443 (52.7)	
Right	382 (46.3)	
T stage		<0.001
T1	56 (6.8)	
T2	380 (46.0)	
T3	188 (22.8)	
T4	201 (24.4)	
N stage		<0.001
N0	732 (88.7)	
N1	93 (11.3)	
M stage	–	<0.001
M0	541 (65.6)	
M1	284 (34.4)	
Liver metastasis		<0.001
Yes	152 (18.4)	
No	673 (81.6)	
Lung metastasis		<0.001
Yes	187 (22.7)	
No	638 (77.3)	
Bone metastasis		<0.001
Yes	63 (7.6)	
No	762 (92.4)	
Tumor size (mm)		<0.001
>85	554 (67.1)	
≤85	271 (22.9)	
Surgery		<0.001
Yes	649 (78.6)	
No	176 (21.4)	

SEER, Surveillance, Epidemiology, and End Results.

**Figure 4:**
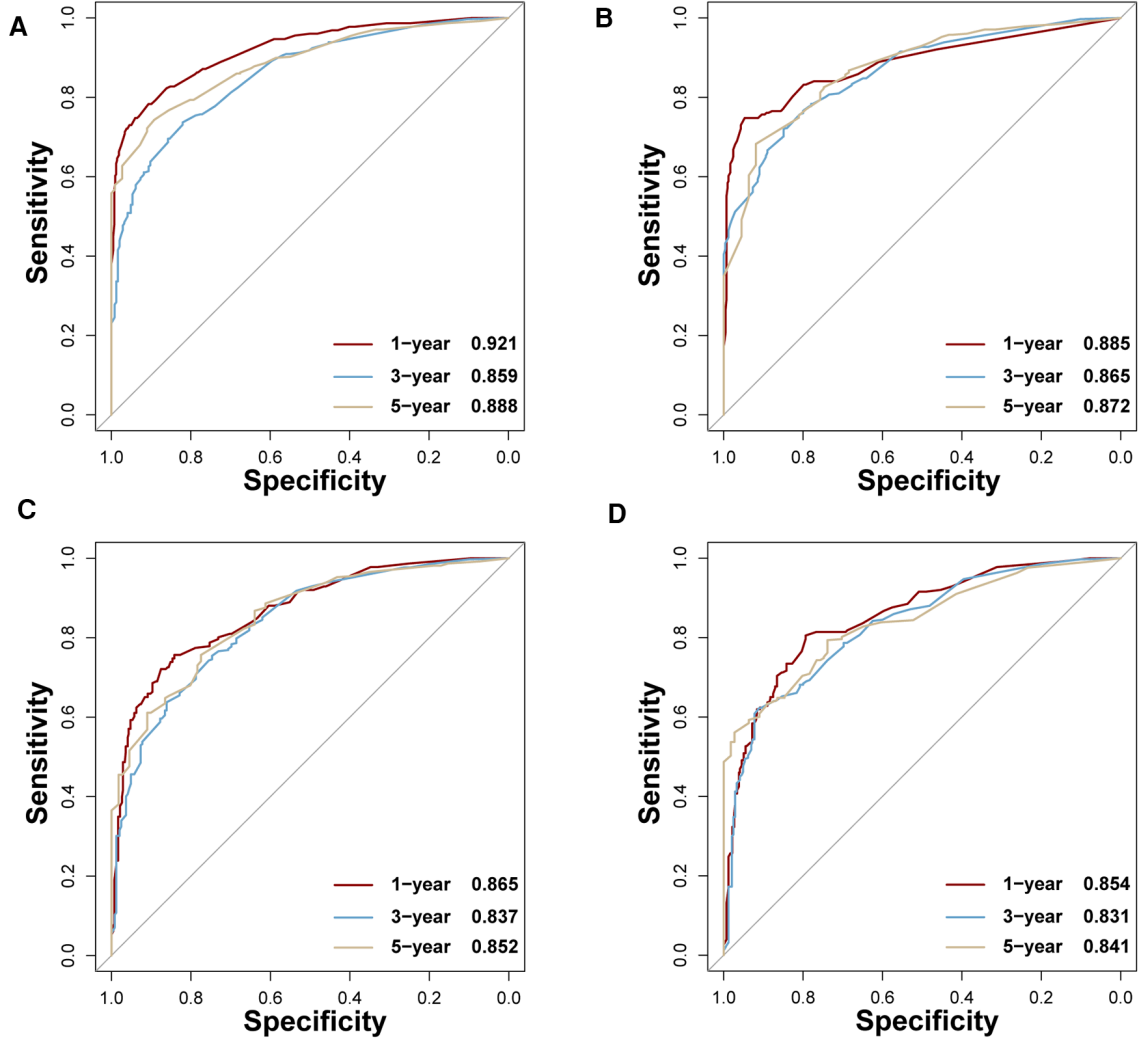
AUCs of four ML-based models for the training set at 1-, 3-, and 5-year points. (**A**) BP-ANN, (**B**) RF, (**C**) SVM, (**D**) NBC.

**Figure 5:**
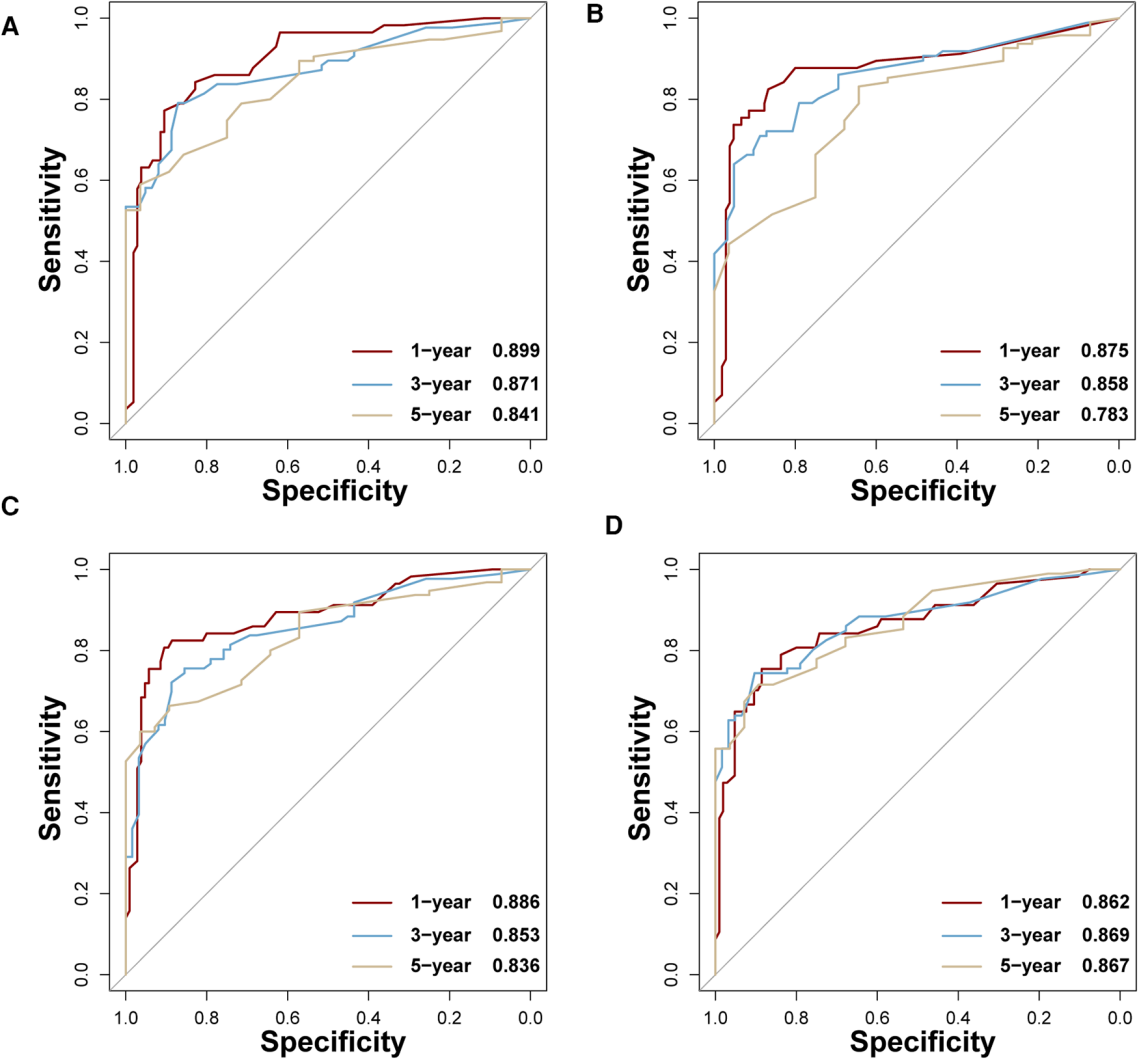
AUCs of four ML-based models for the test set at 1-, 3-, and 5-year points. (**A**) BP-ANN, (**B**) RF, (**C**) SVM, (**D**) NBC.

In the training set, the AUCs of BP-ANN, RF, SVM, and NBC for predicting 1-year survival status were 0.921, 0.885, 0.865, and 0.854; those for predicting 3-year survival status were 0.859, 0.865, 0.837, and 0.831; those for 5-year survival status were 0.888, 0.872, 0.852, and 0.841, respectively (Figure 4). In the test set, the AUCs of these four models for 1-year survival status were 0.899, 0.875, 0.886, and 0.862; those for 3-year status were 0.871, 0.858, 0.853, and 0.869; and those for 5-year status were 0.841, 0.783, 0.836, and 0.867, respectively (Figure 5). It was apparent that AUROCs of BP-ANN were at higher than expected values for the 3-year point in the training set and the 5-year point in the test set. In addition, the results of RF and NBC were superior to those of BP-ANN (0.865 vs. 0.859; 0.867 vs. 0.841) (Table 3).

**Table 3:** Comparison of AUROCs among four ML-based models in the training set and test set.

–	Algorithms	AUROC (1-year)	AUROC (3-year)	AUROC (5-year)
Training set	BP-ANN	0.921	0.859	0.888
	RF	0.885	0.865	0.872
	SVM	0.865	0.837	0.852
	NBC	0.854	0.831	0.841
Testing set	BP-ANN	0.899	0.871	0.841
	RF	0.875	0.858	0.783
	SVM	0.886	0.853	0.836
	NBC	0.862	0.869	0.867

AUROC, area under the receiver operating characteristic; BP-ANN, backpropagation artificial neural network; RF, random forest; SVM, support vector machine; NBC, naive Bayes classifier.

**Figure 6:**
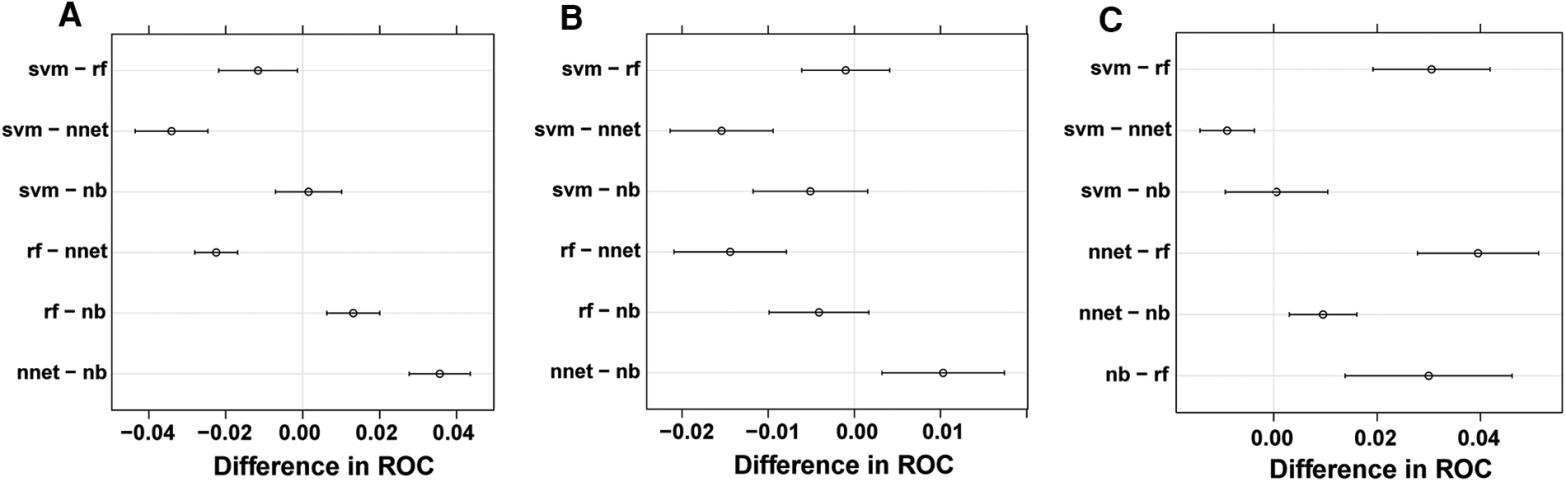
Pairwise model comparison using 10-fold cross-validation method repeated 10 times. (**A**) 1 year, (**B**) 3 years, (**C**) 5 years.

Based upon previous computation, we came to know that BP-ANN might be the best model according to the inner validating method at a ratio of 8 : 2. Sampling errors from the training and test sets may lead to certain distribution characteristics, resulting in a particular model being significantly more or less powerful when compared to other models. To avoid this situation, it was required to determine whether the superiority of BP-ANN was statistically significant. Therefore, a 10-fold cross-validation method repeated 10 times was performed (Table 4). In the process of calculation, all samples were divided into 10 groups of approximately equal size, each one of which was taken as a test set in turn and the other 9 sets were taken to form training sets; this process was repeated a total of 10 times. Each model could generate 10 AUROC values when forecasting the survival status of ACC patients for each time point. After 10 cycles, 100 AUROC values were generated from each model at each time node. The four models produced a total of 12 groups of data, each group consisting of 100 AUROC values. As a result, BP-ANN showed the highest mean AUROCs at all time points among these four ML-based models. By pairwise comparing their efficiencies, averages of 1-, 3-, and 5-year AUROCs of BP-ANN statistically exceeded those of the other three models, and the superiority of BP-ANN was established (Figure 6, *P* < 0.008).

**Table 4:** Results of 10-fold cross-validation method repeated 10 times.

Time point	Algorithm	Averages	Medians
1-year	BP-ANN	0.890	0.894
	RF	0.868	0.871
	SVM	0.856	0.864
	NBC	0.855	0.859
3-year	BP-ANN	0.847	0.856
	RF	0.832	0.836
	SVM	0.831	0.831
	NBC	0.837	0.843
5-year	BP-ANN	0.854	0.862
	RF	0.815	0.829
	SVM	0.845	0.851
	NBC	0.845	0.848

BP-ANN, backpropagation artificial neural network; RF, random forest; SVM, support vector machine; NBC, naive Bayes classifier.

## Discussion

Currently, complete tumor resection (R0 resection) is the only way to cure ACC. The 5-year survival rate of patients receiving R0 resection is approximately 49% (9). When preoperative radiographic examination cannot eliminate the possibility of an adrenal malignant entity, malignancy should be considered or the prognosis of patients should be assumed to be extremely terrible (10). Even if there are no postoperative macroscopic residues, local relapse ranges from 19% to 34% (9, 11). Hence, it is vital to prevent tumor relapse and metastasis for survival of ACC patients. Tumor bed radiotherapy has a long history for ACC patients who have received R1 resection with microscopic residues. However, the results of various research studies regarding adjuvant radiotherapy differ. Fassnacht et al. conducted a study involving 14 ACC patients who underwent radiotherapy and 14 without adjuvant radiotherapy, and they concluded that radiotherapy could significantly decrease the risk for local relapse, but it did not influence metastasis and overall survival (12). Another study involved 16 ACC patients who received and 32 patients who did not receive adjuvant radiotherapy; the results illustrated that radiotherapy could not decrease the risk for local relapse and metastasis and prolong overall survival time (13). The reason for the discrepancy may be a statistical error caused by the small sample size. A large study conducted by Nelson et al., involving 171 ACC patients, also concluded that radiotherapy had no significant effect on local recurrence, distant metastasis, and overall survival rate; however, it reduced the probability of death (14).

In general, owing to the low incidence rate of ACC, large prospective studies supporting the effectiveness of adjuvant radiotherapy are not available. Currently, mitotane is the only drug approved for ACC by the Food and Drug Administration (FDA). It can extend the recurrence-free survival time of ACC patients receiving radical resection surgery (15). At present, scholars have not reached a consensus on whether mitotane should be combined with other cytotoxic drugs. In clinical practice, some medical centers administer patients only with mitotane, while others combine mitotane and platinum complexes. There are different viewpoints on the advantages and disadvantages of the two methods (16).

In addition, previous articles whose data were derived from the SEER database indicated that univariate Cox analysis could not detect an explicit discrepancy between cohorts divided in accordance with “Yes” and “No/Unknown” status of ACC patients. Albeit this may be caused by the uncertainty of “Unknown”, in consideration of the conclusions of the above articles, we believe that it is more likely that radiotherapy and chemotherapy have no significant effect on the overall survival of patients with ACC. The black-box data, “unknown,” of radiotherapy and chemotherapy, the survival analysis of previous scholars, and the results of various clinical trials are reasons why we did not take radiotherapy and chemotherapy into consideration when establishing ML-based models.

In this study, factors involved in ML-based models are already proven to be associated with the prognosis of patients with ACC, such as age, stage, and distant metastasis. In addition, the presence of tumor cells at the edge of the incision, the presence of tumors at the lymph node, neoplastic grading, and hormone secretion by the tumor are also factors that may influence prognosis (16). Secretion of corticosteroids affects the response of ACC to immunotherapy (17). If these elements are added to ML-based models, prediction of patient survival would be more accurate. Unfortunately, these variables were unavailable in the SEER database or were difficult to be statistically evaluated due to incomplete data.

A combination of medicine and artificial intelligence is currently a research hotspot in medicine. Machine-based learning has a huge advantage in processing complex nonlinear data and selecting important features from huge datasets. This provides great assistance in predicting tumor biological behavior (18). Many studies have shown that machine learning offers great value in predicting disease progression. For example, Jiang et al. applied the XGBoost algorithm to predict the 5-year survival status of patients with osteosarcoma, and the AUCs of both training and test sets reached a value of more than 0.9 (19). Alabi et al. compared the capability of the machine learning model and nomogram in predicting the overall survival rate of tongue cancer patients and found that both AUROC and the accuracy of machine learning were higher than the nomogram (20). The key point of this study is to establish a robust and precise ML-based model to predict the survival status of ACC at important time points by using routine clinical indicators. The results show that BP-ANN in the training set is slightly worse than RF in predicting the 3-year survival status, while in the test set, it is slightly weaker than NBC in predicting the 5-year survival status. This result reflects that BP-ANN may be the best choice among the four models when the full SEER dataset was split into training and test sets in 1 : 1 proportion for modeling and internal verification. The subsequent 10-fold cross-validation method repeated 10 times show that the effectiveness of this model is indeed stronger than other models (*p* < 0.008).

This study combines machine learning algorithms with the SEER database to create a survival condition model for patients with ACC for the first time. The disadvantage is that the sample size of patients with ACC collected from the SEER program database is relatively small, which reduces the reliability of validation results. Since it is hard for a single medical center to accumulate a large number of patients with ACC, we expect more public medical databases to provide more detailed clinical data for research.

## Data Availability

The datasets presented in this study can be found in online repositories. The names of the repository/repositories and accession number(s) can be found in the article/Supplementary Material.
